# Articulator Zeroing on Condylar Guidance Values: A Comparative Study of Hanau and Artex Systems

**DOI:** 10.12688/f1000research.179624.1

**Published:** 2026-04-19

**Authors:** Mallikarjuna Ragher, Sanath Kumar Shetty, Vidya Bhat S, Rajesh Shetty, Savitha Dandekeri, Sanha Razdan, Sunaina M Afnan

**Affiliations:** 1 Prosthodontics, Yenepoya (Deemed to be University) Dental College, Mangaluru, Karnataka, India

**Keywords:** Condylar guidance, Facebow Transfer, Interocclusal record, Semi-adjustable articulator, Zeroing.

## Abstract

**Purpose:**

Accurate replication of mandibular movements is essential for successful prosthodontic rehabilitation. Semi-adjustable articulators are commonly used for this purpose, and zeroing is a preparatory step intended to standardize articulator settings. However, its influence on condylar guidance values remains unclear. Therefore the purpose of the study was to evaluate the effect of zeroing on condylar guidance values and compare measurements obtained from Hanau and Artex articulators.

**Methods:**

Thirty dentate participants (20–30 years) were included. Maxillary casts were mounted using facebow transfer, and mandibular casts were mounted in maximum intercuspation. Four groups were evaluated: Hanau with zeroing (H0), Hanau without zeroing (H1), Artex with zeroing (A0), and Artex without zeroing (A1). Condylar guidance was recorded using protrusive interocclusal records. Data were analyzed using statistical tests with significance set at p < 0.05.

**Results:**

Significant differences were found between Hanau and Artex articulators under both zeroing and non-zeroing conditions (p < 0.05). No statistically significant differences were observed within each articulator when comparing zeroing and non-zeroing conditions (p > 0.05).

**Conclusions:**

The difference in the condylar guidance angle between zeroing and non-zeroing of the articulator is minimal and not statistically significant. However, this small variation could potentially impact the accuracy and precision of prosthesis fabrication. Additionally, there is an observable difference in the condylar guidance angle between the Hanau and Artex articulators, when comparing measurements with and without zeroing.

## 1. Introduction

The primary goal of every prosthodontist is to create dental restorations that seamlessly accommodates a patient’s natural functional movements, thereby preventing any disruptions or disharmony within the stomatognathic system. Dental articulators play a pivotal role in accurately replicating these mandibular movements and facilitating the restoration of proper occlusion. Programming semi-adjustable articulators involves adjusting two critical components: horizontal and lateral condylar guidance which are obtained through interocclusal records. The precision and reliability of these programming methods significantly influence the harmony of occlusion. ^1^

The popular articulators feature condylar elements that operate within a slot-type guidance mechanism. These articulators allow for adjustment of horizontal condylar guidance ranging from −40 to +80 degrees, and lateral guidance side shift adjustments ranging from 0 to 20 degrees. ^2^

Before mounting the maxillary cast, all adjustments on the articulator are manually locked without exerting excessive force or mechanical aids. The preparatory step, known as “zeroing” or “articulator preparation,” involves setting specific values for the condylar angle, Bennet angle, and incisal guides as recommended by the manufacturer. 

### 1.1 Manufacturer guidelines for Zeroing

For Hanau Wide Vue articulators, protrusive condylar guidance is set at 30 degrees, lateral condylar guidance at 30 degrees, and incisal guides at zero degrees. The centric locks are tightened to restrict the instrument to opening and closing movements only.

For Artex articulators, protrusive condylar guidance is set at 30 degrees, lateral condylar posts at 15 degrees, and incisal guides at zero degrees. Similarly, the centric locks are tightened to limit the instrument to opening and closing movements only.

Despite its routine use, the clinical significance of zeroing remains debated. Therefore, this study aimed to evaluate the impact of zeroing on condylar guidance values and compare results between Hanau and Artex articulator system.

## 2. Methodology

Institutional ethical approval was obtained from the Yenepoya Ethics Committee-2, Yenepoya (Deemed to be University) (YEC2/564) before the study commenced. Volunteers were enlisted following a comprehensive explanation of the study protocol, and written informed consent was obtained from each participant.

Sample size of 30 was determined based on a significance level of 5% and a standard deviation of 5.99, with a margin of error of 3%. ^5^

Thirty participants aged between 20 and 30 years, with full set of teeth, were included in the study. Exclusion criteria encompassed individuals with periodontally compromised teeth, grossly attrited or abraded teeth, with fixed or removable partial dentures, or missing teeth.

Four sets of maxillary and mandibular impressions were obtained for each of the 30 participants. These impressions were poured in Type III dental stone to create casts and randomly allotted to four groups: Group H0: Hanau with zeroing, Group H1: Hanau non-zeroing, Group A0: Artex zeroing, Group A1: Artex non-zeroing.

**Figure 1.**
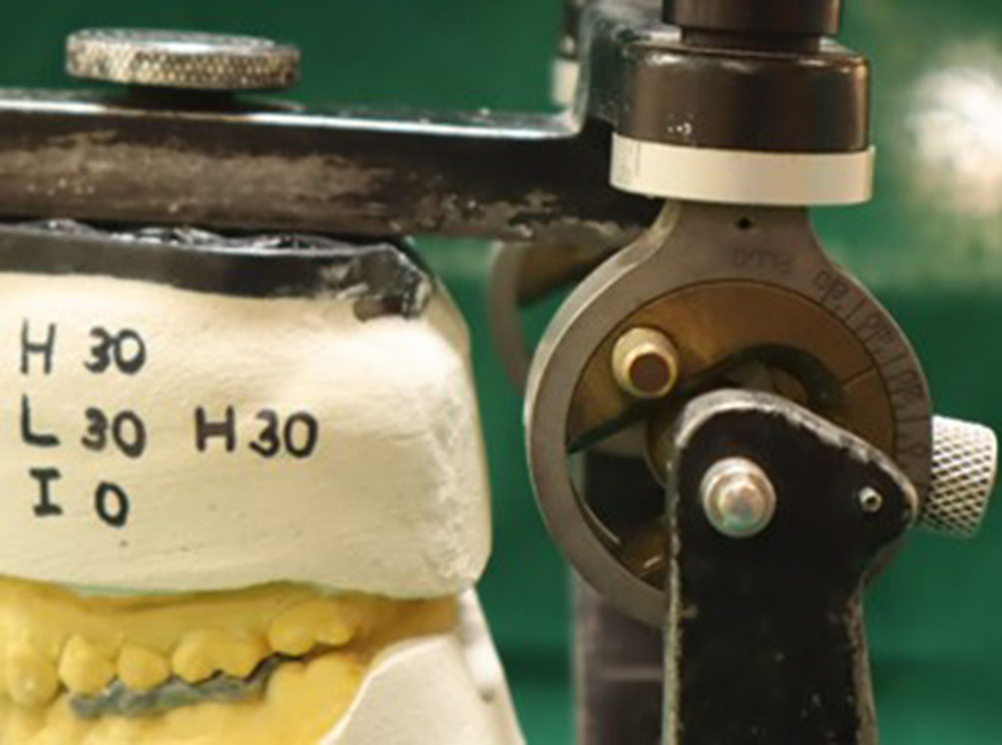
Condylar guidance after zeroing in Hanau.

**Figure 2.**
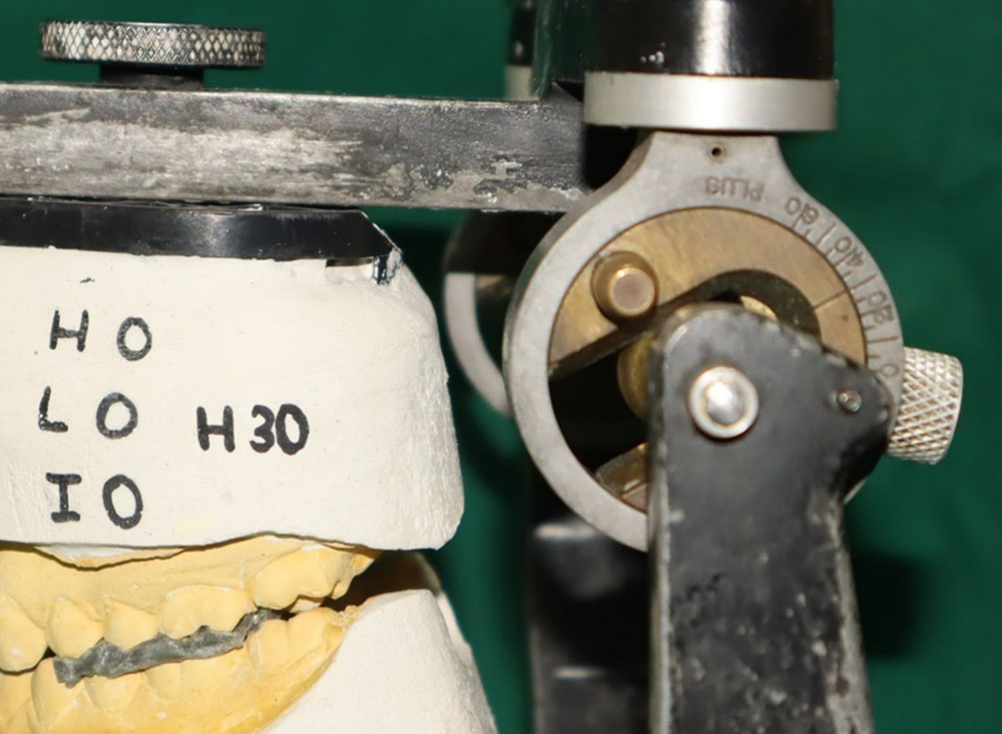
Condylar guidance without zeroing in Hanau.

On the Hanau Wide-Vue articulator, the maxillary casts were mounted using Hanau spring bow with zeroing and non-zeroing settings for each group. In the Hanau zeroing group, the condylar guidance angle was set to 30 degrees, the Bennet angle was set to 30 degrees, and the incisal guidance was set at 0 degrees (zeroing) ( Figure 1). For the Hanau non-zeroing group, the condylar guidance angle was set to 0 degrees, the Bennet angle was set to 0 degrees, and the incisal guidance was also set at 0 degrees ( Figure 2).

**Figure 3.**
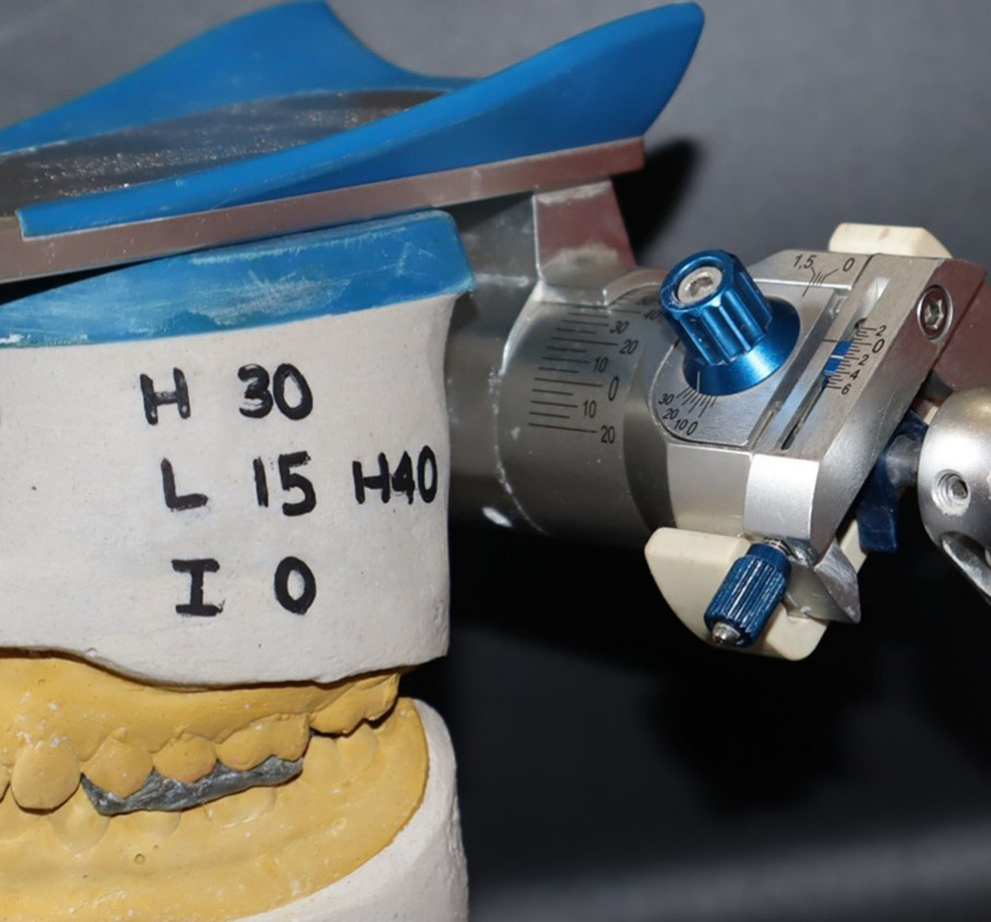
Condylar guidance after zeroing in Artex.

**Figure 4.**
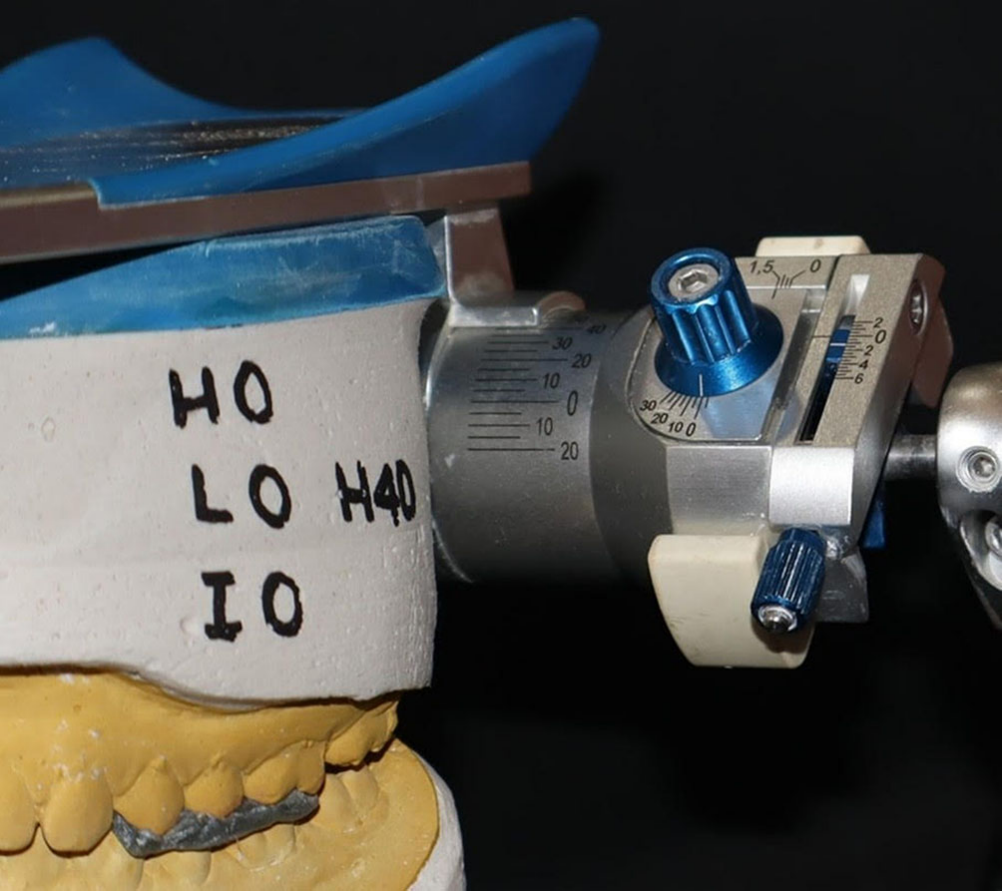
Condylar guidance without zeroing in Artex.

The same procedure was repeated for Artex articulators utilizing the Slidematic Face bow. In the Artex zeroing group, the articulator was set with a condylar guidance angle set to 30 degrees, a Bennet angle set to 15 degrees, and incisal guidance set at 0 degrees ( Figure 3). Conversely, the Artex non-zeroing group’s articulator had values of 0 degrees for the condylar guidance angle, 0 degrees for the Bennet angle, and 0 degrees for incisal guidance ( Figure 4).

All records for each participant were meticulously completed on the same day by the same operator. The mandibular cast was mounted utilizing the patient’s maximum intercuspation record for all articulators. The protrusive interocclusal record was obtained with Aluwax, and these records were employed to program the articulators. The protrusive relation was carefully evaluated and reconfirmed before establishing the sagittal condylar inclinations, with locknuts being securely tightened by hand pressure. All articulators were programmed by a single operator to ensure consistency and accuracy.

Subsequently, the resulting condylar guidance values obtained from each group were meticulously recorded and subjected to statistical analysis. (Supplementary table 1 and 2 – in Data availability repository).

## 3. Results

Data analysis was conducted using SPSS version 27.0 statistical software. The obtained p-values from t-tests indicate significant differences in mean values between the H0 and A0 groups, as well as between the H1 and A1 groups (p < 0.05). These findings suggest distinct mean values between the Hanau (H) and Artex (A) articulators when set at zeroing (0) and non-zeroing (1) positions, respectively.

Conversely, comparisons between the H0 and H1 groups, and between the A0 and A1 groups showed p-values greater than 0.05, indicating no statistically significant differences in mean values within each articulator type across zeroing and non-zeroing settings ( Table 1 and Table 2).

**Table 1.** Comparison of mean condylar guidance values between groups (zeroing vs. non-zeroing and between articulators).

	G	Mean	Std. Deviation	Std. Error Mean
H0A0	H0	35.83	6.309	1.152
	A0	39.83	5.645	1.031
H1A1	H1	35.33	5.713	1.043
	A1	38.50	5.438	0.993
H	H0	35.83	6.309	1.152
	H1	35.33	5.713	1.043
A	A0	39.83	5.645	1.031
	A1	38.50	5.438	0.993

**Table 2.** Independent samples t-test comparing condylar guidance values between articulator groups.

t-test for Equality of Means							
	T	df	p value	Mean Difference	Std. Error Difference	95% Confidence Interval of the Difference	
						Lower	Upper
H0A0	−2.588	58	.012	−4.000	1.546	−7.094	−0.906
H1A1	−2.199	58	.032	−3.167	1.440	−6.049	−0.284
H0H1	.322	58	.749	0.500	1.554	−2.611	3.611
A0A1	.932	58	.355	1.333	1.431	−1.531	4.198

## 4. Discussion

The complexities of mandibular movements necessitate careful consideration when utilizing articulators. Over time, efforts have been made to accurately replicate these movements, resulting in increasingly intricate articulator designs. Semi-adjustable articulators are calibrated using individual static records, but their reliability has often been questioned as they fail to simulate the patient’s temporomandibular joint (TMJ) anatomy and rely on average condylar settings. ^3, 4^

The ability of dentists to consistently zero the instrument before mounting casts is crucial for the proper use of semi-adjustable articulators. This process, known as “zeroing,” involves standardizing the articulator to a consistent starting position. To achieve accurate zeroing, specific criteria must be met, including ensuring that the incisal pin strikes the incisal table centrally in both the antero-posterior and right-to-left directions. Additionally, when the centric locks are engaged, there should be no lateral movement between the upper and lower components. ^5^

Zeroing the articulator positions the condyle at its most anterior superior position within the glenoid fossa, thereby reproducing the most precise guidance at this point. ^6^ According to Hanau manufacturers, zeroing is essential to prevent the articulator from producing flat results that lack the characteristic angulation of a patient’s jaw movement. Typically, most patients’ jaw movements have an average angle of 30 degree. Failure to account for this angulation may result in inadequate contact, particularly with larger arches.

Our study results showed that changing the articulator settings for mounting affects the measured values of condylar guidance. Although we noted a noticeable difference in values between zeroing and non-zeroing positions, the lack of statistical significance suggests that these differences might not be considered meaningful in a statistical sense ( Table 2).

However, the fact that there are noticeable differences implies that these adjustments could still have practical implications, particularly in prosthesis fabrication where precise measurements are crucial. Even though the statistical test did not show significance, the consistent differences observed could lead to errors if not properly accounted for during prosthesis fabrication.

The statistical difference between the Hanau and Artex articulators within both the zeroing and non-zeroing groups may be linked to differences in how each articulator defines or utilizes the anterior reference point.

Articulators rely on specific reference points to simulate jaw movements and occlusal relationships accurately. These reference points, especially the anterior reference point, play a crucial role in determining the overall articulator settings and consequently affect measurements such as condylar guidance values.

The Hanau and Artex articulators may have distinct methods or positions for establishing the anterior reference point. This variation can lead to differences in how condylar guidance values are interpreted and measured between the two articulator types, potentially resulting in statistically significant discrepancies as observed in your study.

Therefore, when comparing data between these articulators, it’s essential to consider not only the settings like zeroing or non-zeroing but also how each articulator system defines and utilizes critical reference points such as the anterior reference point. These differences can significantly impact the outcomes and conclusions drawn from studies involving dental articulation and occlusal analysis. This understanding could help in refining protocols or methodologies to minimize errors and ensure more accurate results in clinical practice.

## 5. Conclusion

Based on our findings, we conclude that adjusting articulator settings, whether zeroing or non-zeroing, despite not achieving statistical significance, shows practical implications due to observable differences. Additionally, there is an observable difference in the condylar guidance angle between the Hanau and Artex articulators, when comparing measurements with and without zeroing. In prosthesis fabrication, where precision is paramount, these adjustments have the potential to introduce errors. Therefore, despite the statistical tests not confirming significance, the consistent variations observed necessitate further investigation. It is crucial to thoroughly understand these effects to refine protocols aimed at ensuring precise clinical outcomes.

## Data Availability

IMPACT OF ZEROING OF ARTICULATORS ON CONDYLAR GUIDANCE ANGLE [Data set]. Zenodo. https://doi.org/10.5281/zenodo.19247183 ^7^ The repository contains- supplementary tables, informed consent form and participant information sheet. Data are available under the terms of the Creative Commons Attribution 4.0 International license (CC-BY 4.0).
