# Supplementary material for: Articulator Zeroing on Condylar Guidance Values: A Comparative Study of Hanau and Artex Systems
Source: F1000Res. 2026 Apr 19;15:580. [Version 1] doi: 10.12688/f1000research.179624.1 (PMC13173281; doi:10.12688/f1000research.179624.1)
Supplement: Peer review history

# Reviewer report: Reviewer response for version 1

**DOI:** 10.5256/f1000research.198156.r478354

**Published:** 2026-05-13

**Authors:** Mukesh Kumar Goyal

**Affiliations:** 1 PROSTHODONTICS, Inderprastha Dental College & Hospital, Ghaziabad, Uttar Pradesh, India

Dear Authors,

 Same study has been cited as reference no. 7 by same authors.

 Topic selected is very controversial in the literature and in practical application. It is a great effort to overcome by this type of study. This study is definitely helpful to overcome the controversy in the application of articulators whether it needs zeroing or not.

 1. Key word "Zeroing" need to modify as "Zeroing of Articulators"

 2. In the abstract need to mention use of type of Inter-occlusal records.

 3. In introduction " Therefore, this study aimed to evaluate the impact of zeroing on condylar guidance values and compare results between Hanau and Artex articulator system" add zeroing of articulators and compare values of horizontal condylar guidance between .....

 4. How the Sample size was decided. what statistical analysis was performed to calculate sample size?

 5. Inclusion and Exclusion criteria are incomplete. Need to mention about TMJ disorders, neurological Disorders etc. 

 6.  With which material initial impressions were made .. not mentioned and why four sets.. it could have been done with 2 sets.

 7. Need to mention which company or brand Type III stone was used?

 8. In Figure 1 & 2 do not show maximum intercuspation. How did you manage that occlusal separation?

 9. Now a days More accurate and advanced materials are available for interocclusal records which are rigid enough to duplicate CG,and does not get distorted. Why have you not used elastomeric interocclusal record materials?

 10. in the result part mean values need to be mentioned for each group in the text.

 11. As comparing two articulators, results are highly significant and variations exist. There could have been some standardization to which both articulators can be compared, and conclusion can be made which articulator is more accurate and closer to actual reading of patient.

 12. No recent references have been added. Only reference no. 7 is of 2026 .. which looks same study and same authors

Is the work clearly and accurately presented and does it cite the current literature?

No

If applicable, is the statistical analysis and its interpretation appropriate?

I cannot comment. A qualified statistician is required.

Are all the source data underlying the results available to ensure full reproducibility?

Yes

Is the study design appropriate and is the work technically sound?

Yes

Are the conclusions drawn adequately supported by the results?

Yes

Are sufficient details of methods and analysis provided to allow replication by others?

Yes

Reviewer Expertise:

PROSTHODONTICS

I confirm that I have read this submission and believe that I have an appropriate level of expertise to confirm that it is of an acceptable scientific standard.

# Reviewer report: Reviewer response for version 1

**DOI:** 10.5256/f1000research.198156.r478358

**Published:** 2026-04-28

**Authors:** Rajesh Sethuraman

**Affiliations:** 1 K M Shah Dental College and Hospital, Sumandeep Vidyapeeth, Vadodara, Gujarat, India

The study focuses on a concept that has less to nil literature available. Articulator setting have always been followed as suggested by the manufacturer. However, the evidence to support these zeroing guidelines does not exist.

 The aim of the study was to evaluate the effect of articulator zeroing on values of saggital condylar inclination. The results therefore could be extrapolated only to Protrusive angulation. Study focus could also be on Lateral condylar inclination and evaluation if the inclination values are different to those obtained by radiographic means. The sample size calculations were probably made from the article by [Nowlin T, Bailey J, Taubert **Accuracy of zeroing the articulator. **The Journal of Prosthetic Dentistry, 57, 647-649.] Though this reference study was on zeroing, I would still advise the current study as a pilot observation and to expand it further with a robust sample size and gold standard comparator. The methodology and statistical analysis used in the study seem adequate and justifiable. 

 In the dearth of similar studies, the discussion can only focus on the current study. Clinically significance could be explained with literature established values that can add more value to the results obtained.

Is the work clearly and accurately presented and does it cite the current literature?

Yes

If applicable, is the statistical analysis and its interpretation appropriate?

Yes

Are all the source data underlying the results available to ensure full reproducibility?

Yes

Is the study design appropriate and is the work technically sound?

Yes

Are the conclusions drawn adequately supported by the results?

Yes

Are sufficient details of methods and analysis provided to allow replication by others?

Yes

Reviewer Expertise:

Removable Prosthodontics, Fixed Prosthodontics, Dental Material Sciences, Implant Prosthodontics, Maxillofacial Prosthodontics, Digital Prosthodontics.

I confirm that I have read this submission and believe that I have an appropriate level of expertise to confirm that it is of an acceptable scientific standard.
